# Synthesis, Characterization, Anti-Cancer Analysis of Sr_0.5_Ba_0.5_Dy*_x_*Sm*_x_*Fe_8−2*x*_O_19_ (0.00 ≤ *x* ≤ 1.0) Microsphere Nanocomposites

**DOI:** 10.3390/nano11030700

**Published:** 2021-03-11

**Authors:** Suhailah S. Al-Jameel, Munirah A. Almessiere, Firdos A. Khan, Nedaa Taskhandi, Yassine Slimani, Najat S. Al-Saleh, Ayyar Manikandan, Ebtesam A. Al-Suhaimi, Abdulhadi Baykal

**Affiliations:** 1 Department of Chemistry, College of Science, Imam Abdulrahman Bin Faisal University, P.O. Box 1982, Dammam 31441, Saudi Arabia; ssaljameel@iau.edu.sa; 2 Department of Biophysics, Institute for Research and Medical Consultations (IRMC), Imam Abdulrahman Bin Faisal University, P.O. Box 1982, Dammam 31441, Saudi Arabia; malmessiere@iau.edu.sa (M.A.A.); yaslimani@iau.edu.sa (Y.S.); 3 Department of Stem Cell Research, Institute for Research and Medical Consultations (IRMC), Imam Abdulrahman Bin Faisal University, P.O. Box 1982, Dammam 31441, Saudi Arabia; 4 Department of Nanomedicine Research, Institute for Research and Medical Consultations (IRMC), Imam Abdulrahman Bin Faisal University, P.O. Box 1982, Dammam 31441, Saudi Arabia; natashkandi@iau.edu.sa (N.T.); abaykal@iau.edu.sa (A.B.); 5 Consultant Family and Community Medicine, Imam Abdulrahman Bin Faisal University, P.O. Box 1982, Dammam 31441, Saudi Arabia; nalsaleh@iau.edu.sa; 6 Department of Chemistry, Bharath Institute of Higher Education and Research (BIHER), Bharath University, Chennai 600 073, Tamil Nadu, India; manikandana.che@bharathuniv.ac.in; 7 Biology Department, Science College, Imam Abdulrahman Bin Faisal University, P.O. Box 1982, Dammam 31441, Saudi Arabia; ealsuhaimi@iau.edu.sa

**Keywords:** synthesis, hexaferrites, anti-cancer activity, microsphere nanoparticles, confocal microscopy

## Abstract

There is enormous interest in combining two or more nanoparticles for various biomedical applications, especially in anti-cancer agent delivery. In this study, the microsphere nanoparticles were prepared (MSNPs) and their impact on cancer cells was examined. The MSNPs were prepared by using the hydrothermal method where strontium (Sr), barium (Ba), dysprosium (Dy), samarium (Sm), and iron oxide (Fe_8−2*x*_O_19_) were combined, and dysprosium (Dy) and samarium (Sm) was substituted with strontium (Sr) and barium (Ba), preparing Sr_0.5_Ba_0.5_Dy*_x_*Sm*_x_*Fe_8−2*x*_O_19_ (0.00 ≤ *x* ≤ 1.0) MSNPs. The microspheres were characterized by X-ray powder diffraction (XRD), high-resolution transmission electron microscopy (HR-TEM), transmission electron microscopy (TEM), scanning electron microscopy (SEM), and energy-dispersive X-ray spectroscopy (EDX) techniques. The diffraction pattern of nanohexaferrites (NHFs) reflected the signature peaks of the hexagonal structure. The XRD revealed a pure hexagonal structure without any undesired phase, which indicated the homogeneity of the products. The crystal size of the nanoparticles were in the range of 22 to 36 nm by Scherrer’s equation. The SEM of MSNPs showed a semi-spherical shape with a high degree of aggregation. TEM and HR-TEM images of MSNPs verified the spherical shape morphology and structure that approved an M-type hexaferrite formation. The anti-cancer activity was examined on HCT-116 (human colorectal carcinoma) and HeLa (cervical cancer cells) using MTT (3-(4,5-Dimethylthiazol-2-yl)-2,5-diphenyltetrazolium bromide) assay and post-48 h treatment of MSNPs caused a dose-dependent inhibition of HCT-116 and HeLa cell proliferation and growth. Conversely, no significant cytotoxic effect was observed on HEK-293 cells. The treatments of MSNPs also induced cancer cells DNA disintegration, as revealed by 4′,6-diamidino-2-phenylindole (DAPI) staining. Finally, these findings suggest that synthesized MSNPs possess potential inhibitory actions on cancerous cells without harming normal cells.

## 1. Introduction

Nanomaterials are promising materials for various biomedical applications, including diagnosis and treatment of cancers. These nanomaterials possess unique properties like high surface area and better cancer cell penetration capability [1]. Inorganic nano-based carriers have been used in drug delivery in different types of cancer cells because of their unique and versatile characteristics such as better biocompatibility, effective and precise cancer cell penetration [2,3,4,5]. Many inorganic nanomaterials such as carbon materials, iron oxide, calcium phosphate, gold, and silicon oxide have been used in many applications [6,7,8,9,10,11,12]. Many issues are still associated with inorganic nano-based carriers, such as high leakage of drugs, non-biodegradability, and low cellular internalization, which all affect drug delivery. New and better strategies are required to overcome these issues. These issues can be resolved by combining two or more nanoparticles, and previously we have shown that the combination of two or more nanoparticles is effective strategies for targeted drug delivery and cancer treatment [13,14,15,16]. A combination of different nanoparticles has been reported to concurrently target cancer cells, and tumors-related inflammation is an efficient anti-tumor approach [17]. 

Individually, Sr, Ba, Dy, Sm, Fe_8−2x_O19, and Ba elements showed biological activities. For example, Sr is used in the development of drug strontium ranelate for osteoporosis treatment [18,19]. There are reports that strontium nanoparticles are used in cancer cell detection and treatment [12,20]. Barium nanoparticles have been used as drug carriers and treating cancer cells [21,22,23,24]. Dysprosium nanoparticles have been used for targeted cancer imaging and chemotherapy [25,26]. Samarium nanoparticles have also been reported to enhance anti-cancer activity [27] and deliver anti-cancer drugs [28]. Iron oxide nanoparticles are also being used for cancer cell imaging, and as nanocarriers to deliver anti-cancer drugs and treat cancer [29,30,31,32,33]. Considering this combination approach, for the first time, we have prepared nanocomposites, where the elements of Sr, Ba, Dy, Sm, and Fe_8−2x_O19 were combined, and Dy, Sm was substituted with Sr, Ba, and prepared Sr_0.5_Ba_0.5_Dy*_x_*Sm*_x_*Fe_8−2x_O_19_ (0.00 ≤ *x* ≤ 1.0) MSNPs by hydrothermal approach. The microspheres were evaluated by using XRD, TEM, SEM, and EDX techniques. The impact of Sr_0.5_Ba_0.5_Dy*_x_*Sm*_x_*Fe_8−2x_O_19_ (0.00 ≤ *x* ≤ 1.0) MSNPs was examined using MTT (3-(4,5-Dimethylthiazol-2-yl)-2,5-diphenyltetrazolium bromide) assay on HCT-116 and HeLa in comparison with HEK-293 (embryonic kidney cells) as a healthy cell line. 

## 2. Materials and Methods

### 2.1. Chemicals and Instrumentations

Sr(NO_3_)_2_ (96%), Ba(NO_3_)_2_ (96%), Fe(NO_3_)_3_.9H_2_O (98%), Dy(NO_3_)_3_.H_2_O (99%), Sm(NO_3_)_3_.4H_2_O (99%) and glucose (C₆H₁₂O₆) were received from Merck and used without any further purification. The phase identification of microsphere compositions was accomplished by Rigaku Benchtop Miniflex XRD analyzer, Japan (Cu Kα radiation at room temperature, 2*θ* = 20 − 70° with 2*θ* = 15 − 75°, step 0.02 (deg) and 5.0 (deg/min)). The surface analysis of the samples was completed by SEM-EDX analysis performed using an (SEM, FEI Titan ST with EDX, Notre Dame, IN, USA) equipped with an EDX detector of EDAX. The SEM imaging was carried out using a beam setting of 5 keV of acceleration voltage and 0.1 nA of probe current. Secondary electron (SE) and backscattered electron (BE) signals were collected simultaneously by using two in-lens detectors, T1 for backscattered and T2 for secondary. In particular, SE signals revealed the topographical features of samples and the BE signals those compositional. For EDX analysis a different beam setting was applied, 20 keV of acceleration voltage and 0.8 nA of probe current, to have a good signal-to-noise ratio, and collect the X-ray signals of heavier elements. The TEM imaging was performed using (FEI, Morgagni 268, FEI Compnay, Prague, Czech Republic) equipped with a field emission gun operated at 300 keV. The TEM samples were prepared by grinding the powder material in an agate mortar and then dispersing the fine powder using ethanol; afterward, a few microliters of the as-obtained suspension were deposited on carbon-coated TEM grids. TEM (HRTEM) observations were acquired by using a low-background double-tilt holder and exhibited a polycrystalline nature of both samples.

#### Process of Synthesis and Characterization of Sr_0.5_Ba_0.5_Dy*_x_*Sm*_x_*Fe_8−2x_O_19_ (0.00 ≤ *x* ≤ 1.0)

Firstly, carbon microsphere template was fabricated by dissolving 1M of glucose in Deionized (DI) water at 40 °C then shifted to a Teflon-lined autoclave at 180 °C for 10 h. Next, the mixture was liquidated and rinsed with hot water few times, then dried at 200 °C for 2 h to obtain a black powder. The Sr_0.5_Ba_0.5_Dy*_x_*Sm*_x_*Fe_8−2x_O_19_ (0.00 ≤ *x* ≤ 1.0) MSNPs was produced hydrothermally. A precise ratio of Sr(NO_3_)_2_, Ba(NO_3_)_2_, Fe(NO_3_)_3_.9H_2_O, Dy(NO_3_)_3_.6(H_2_O), Sm(NO_3_)_3_.6(H_2_O) and 1g of carbon microsphere were thawed in 200 mL of DI water under stirring for 30 min. Ammonia solution was added to adjust the pH 7 of the solution through stirring for 30 min and then proceeded to sonication for 30 min. Further, the mixture was put in a Teflon-lined autoclave at 180 °C for 10 h. Eventually, the solution was dried, grinded and placed in a furnace at 500 °C for 4 h. 

### 2.2. Anti-Cancer Activity

#### 2.2.1. Cell Culture and Testing of MSNPs 

Cancer cell lines such as HCT-116 and HeLa were considered to evaluate the impact of MSNPs on cancer cell viability and cancer cell proliferation. Non-cancer cell line, HEK-293 was used as control cell line and to examine the specificity of the MSNPs. As per previously described method [34,35], the cells were cultured and maintained in the DMEM media, L-glutamine (5%), penicillin (1%), streptomycin (1%), FBS (10%) and selenium chloride (1%) in (5%) a CO_2_ incubator (Thermo-Fisher Scientific, Inc., Waltham, MA, USA) at 37 °C. The cells were seeded in 96 well culture plates and they reached 75–80% confluence. They were processed for testing samples using MTT assay (Baig et al., 2020; Rehman et al., 2020). The MTT assay was done as per the previous study and the (HCT-116, HeLa and HEK-293, ATCC, Manassas, VA, USA) cells were treated with MSNPs with dosages ranging from 5.0 µg to 75 µg/mL. The cells were treated for 48 h and processed for MTT assay. In the control group, MSNPs were not added. Both the control and MSNP-treated cells were exposed to 10 µL of MTT (5 mg/mL) and were incubated in a CO_2_ incubator for 4 h. After that, cell culture media was replaced with DMSO (1%), and the 96-well plate was then examined under a Plate reader (Bio-Tek Instruments, Winooski, VT, USA) at a wavelength of 570 nm. The data obtained from triplicates and one-way ANOVA were followed by Dennett’s post hoc test with Graph-Pad Prism Software (version 6.0 GraphPad Software, San Diego, CA, USA) for final statistical analysis.

#### 2.2.2. DAPI Staining for DNA Analysis

A simple-to-use fluorescent stain, 4′,6-diamidino-2-phenylindole (DAPI), visualizes nuclear DNA in both living and fixed cells. DAPI staining was used to determine the number of nuclei and to assess gross cell morphology. MSNP-treated cancer cells were examined by DAPI staining assay. In the control group, MSNPs were not added, whereas in the experimental group, Sr_0.5_Ba_0.5_Dy*_x_*Sm*_x_*Fe_8−2x_O_19_ (0.00 ≤ *x* ≤ 1.0) MSNPs (50 µg/mL) was added. Post 48h treatment, both groups were treated with (4%) paraformaldehyde and followed by Triton X100-PBS (phosphate-buffered saline) wash. Then cells were stained with DAPI (1.0 μg/mL) under a dark environment for 5 min. The DNA staining was examined by using Confocal Scanning Microscope (Zeiss, Berlin, Germany). 

## 3. Results and Discussion

### 3.1. Structure and Morphology of MSNPs

**Figure 1:**
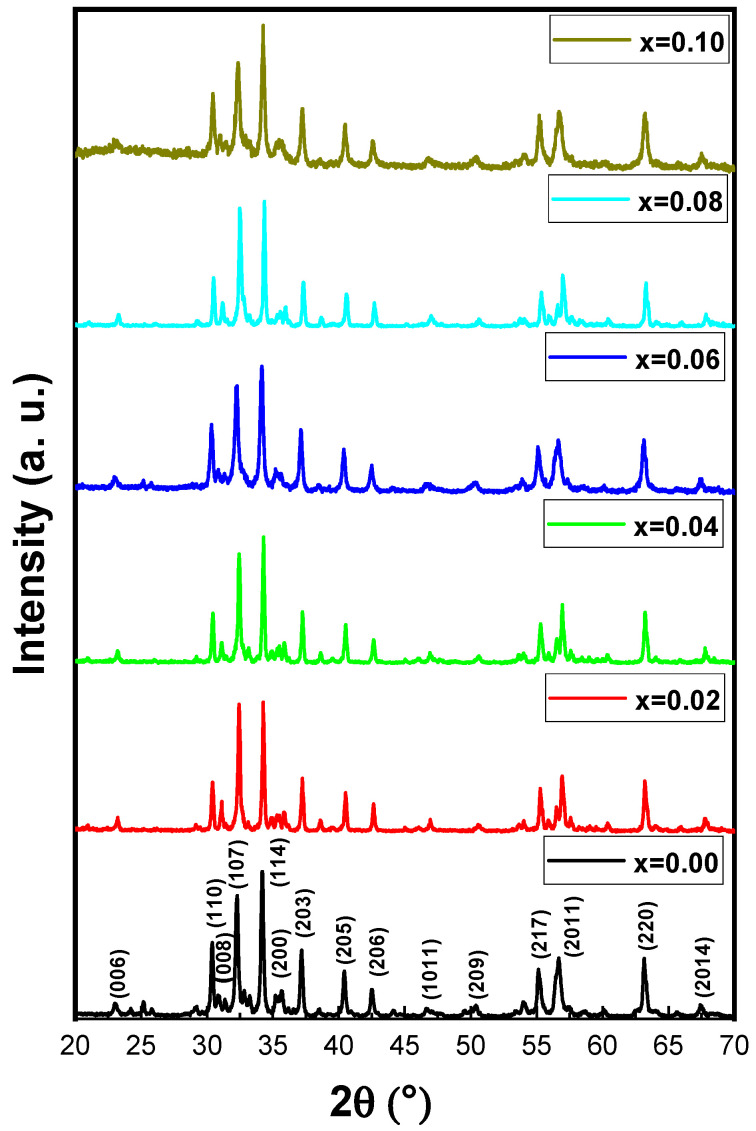
XRD powder patterns of Sr_0.5_Ba_0.5_Sm*_x_*Dy*_x_*Fe_8−2x_O_19_ (0 ≤ *x* ≤ 0.10) MSNPs.

**Figure 2:**
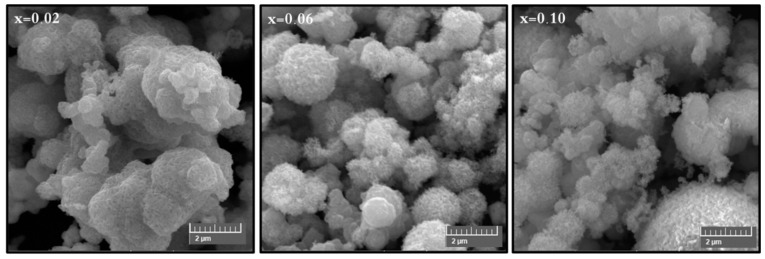
SEM images of Sr_0.5_Ba_0.5_Sm*_x_*Dy*_x_*Fe_8−2x_O_19_ (*x* = 0.02, 0.06 and 0.10) MSNPs.

**Figure 3:**
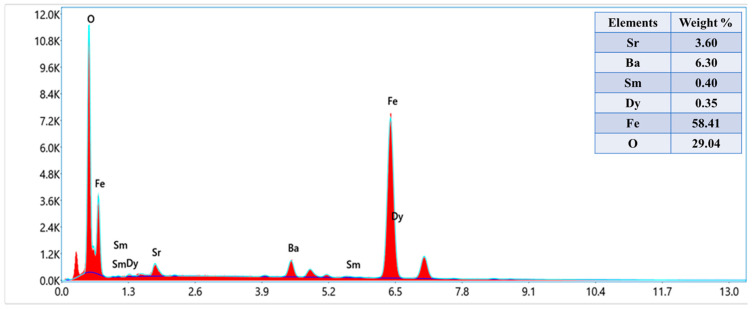
Energy-dispersive X-ray spectroscopy (EDX) spectrum of Sr_0.5_Ba_0.5_Sm_x_Dy_x_Fe_8−2x_O_19_ (*x* = 0.02) MSNPs.

**Figure 4:**
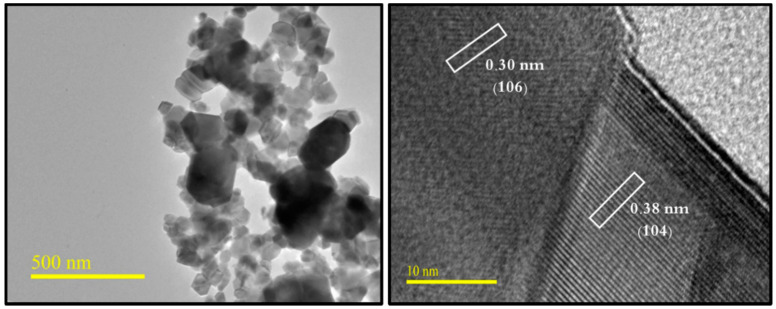
TEM and HR-TEM micrographs of Sr_0.5_Ba_0.5_Sm*_x_*Dy*_x_*Fe_8−2x_O_19_ (*x* = 0.02) MSNPs.

The phase investigation of Sr_0.5_Ba_0.5_Dy*_x_*Sm*_x_*Fe_8−2x_O_19_ (0.00 ≤ *x* ≤ 1.0) MSNPs was proceeded via X-ray diffractometer as presented Figure 1. The diffraction pattern of nanohexaferrites (NHFs) reflected the signature peaks of hexagonal structure. Furthermore, the XRD revealed a pure hexagonal structure without any undesired phase which indicated the homogeneity of the product. The structure parameters a, b and c were estimated through Match 3! Software and it found that they increased with increasing the ratio of Dy and Sm, such as a = b = 5.883(8) − 5.888(6) (Å) and c = 23.156(6) − 23.161(6) (Å) respectively. The crystal size of the products was valued by Scherrer’s equation and found as 34, 24, 22, 31, 30 and 36 nm, respectively. Figure 2 presents the SEM images of Sr_0.5_Ba_0.5_Sm*_x_*Dy*_x_*Fe_8−2x_O_19_ (*x* = 0.02, 0.06 and 0.10) MSNPs. The products were shown the semi-spherical shape with high degree of aggregation. The EDX of Sr_0.5_Ba_0.5_Sm*_x_*Dy*_x_*Fe_8−2x_O_19_ (*x* = 0.02) MSNP are revealed in Figure 3. The EDX spectrum offered the weight percentage of the contained elements as Sr, Ba, Sm, Dy, O and Fe without existence to any impurity. Additionally, Figure 4 unveils the TEM and HR-TEM images of Sr_0.5_Ba_0.5_Sm*_x_*Dy*_x_*Fe_8−2x_O_19_ (*x* = 0.02) MSNP. These images verified the spherical shape morphology and structure that approved the formation of M-type hexaferrite with particle size around 40 nm.

### 3.2. Anti-Cancer Activity

#### 3.2.1. Impact of MSNPs on Various Cancer Cell Lines 

**Figure 5:**
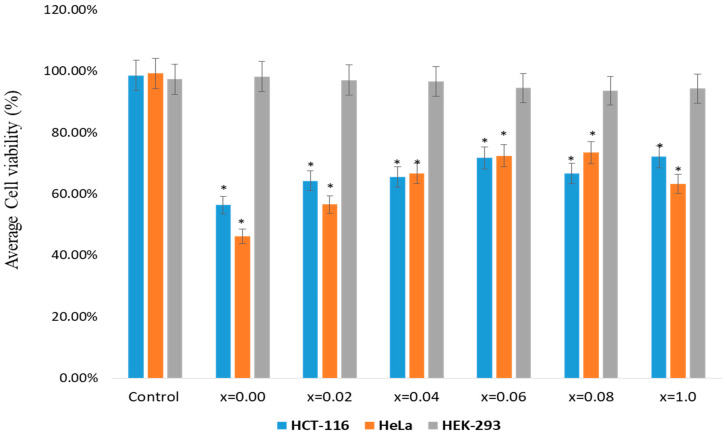
Cell viability by using MTT (3-(4,5-Dimethylthiazol-2-yl)-2,5-diphenyltetrazolium bromide) assay: The MTT assay was done on HCT-116, HeLa and HEK-293 cells which were treated with Sr_0.5_Ba_0.5_Dy*_x_*Sm*_x_*Fe_8−2x_O_19_ (0.00 ≤ *x* ≤ 1.0) MSNPs with dosages ranging from 5.0 µg to 75 µg/mL. The cells were treated for 48 h and processed for MTT assay. In the control group, MSNPs was not added. * *p* < 0.05.

The impact of MSNPs on colon cancer (HCT-116) and cervical cancer (HeLa) cells was examined. The cell viability assay confirmed a significant decrease in the cell viability after the treatments of MSNPs. The treatments of MSNPs showed inhibitory action on cancer cell growth and proliferation (Figure 5).

The inhibitory concentration (IC_50_) of MSNPs were calculated as 56 to 72 µg/mL for HCT-116 and 46 to 63 µg/mL for HeLa cells. We have also tested the impact of MSNPs on non-cancerous cells (HEK-293), though there was minor decrease in the cancer viability, but the percentage of decrease was not statistically significant. Based on these observations, we may suggest that synthesized MSNPs possess inhibitory effect on HCT-116 and HeLa cells than HEK-293 cells. This is the first study demonstrating the cell viability of MSNPs against HCT-116 and HeLa cells. We have previously reported the impact of different nanomaterials on colon and breast cancer cells [36,37,38]. 

#### 3.2.2. Disintegration of Cancer DNA 

**Figure 6:**
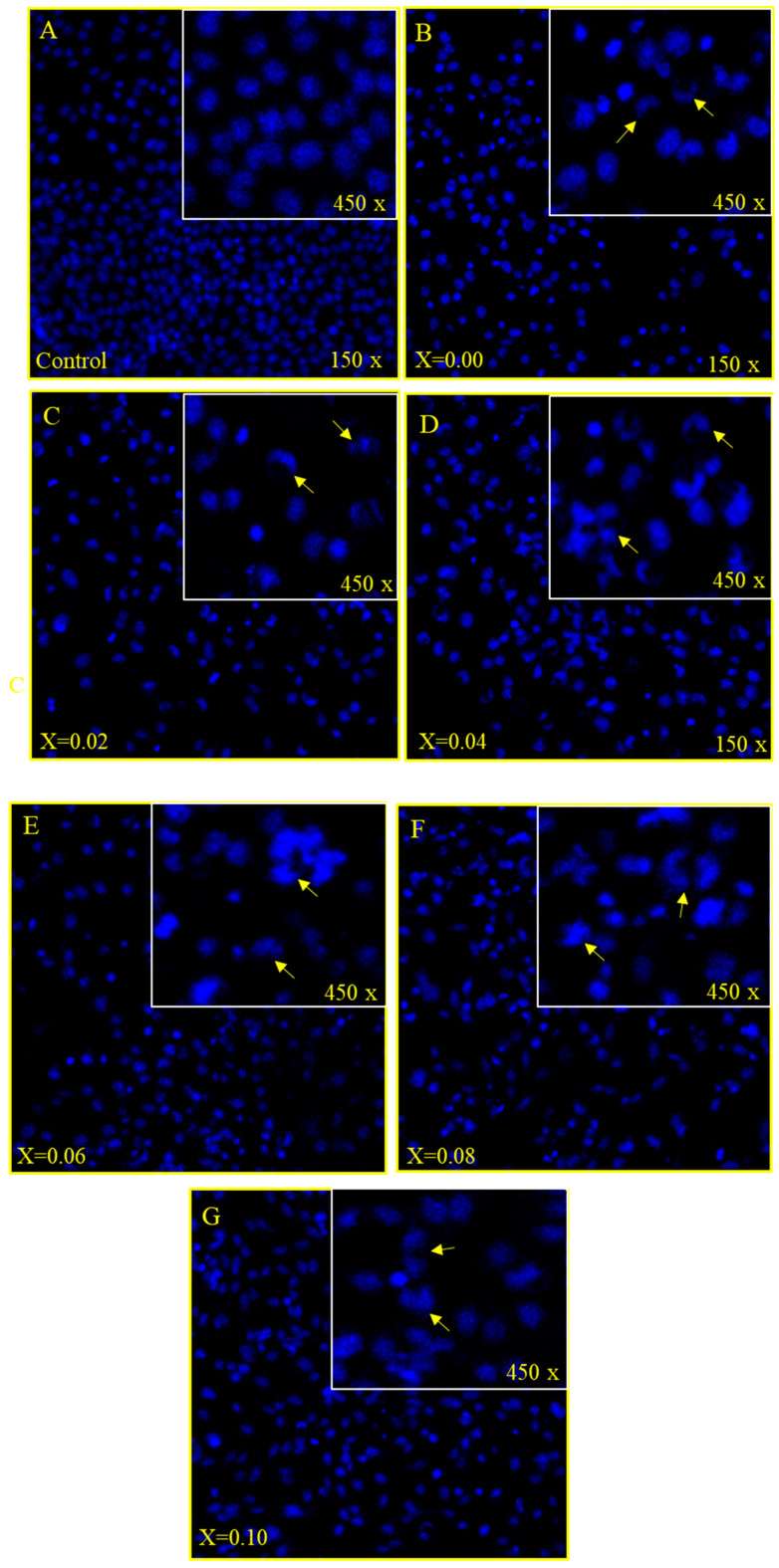
(**A**–**D**): Cancer cell death due treatment of nanoparticles, showing the impact of treatment of nanoparticles on HCT-116 cells stained with DAPI post 48 h treatment. (**A**) is the control cell alongside (**B**) (*x* = 0.00), (**C**) (*x* = 0.02), (**D**) (*x* = 0.04) where a significant number of cancer cells death can be observed due to (50 µg/mL) treatment. Arrows show the cell membrane disruption, nuclear condensation and fragmentation. (**E**–**G**): Cancer cell death due treatment of nanoparticles, showing the impact of treatment of nanoparticles on HCT-116 cells stained with DAPI post 48 h treatment. (**A**) is the control cell alongside (**E**) (*x* = 0.06), (**F**) (*x* = 0.08), (**G**) (*x* = 0.10) where a significant number of cancer cells death can be observed due to (50 µg/mL) treatment. Arrows show the cell membrane disruption, nuclear condensation and fragmentation.

The treatment of MSNPs caused significant morphological and cytological changes in the cancer cells as MSNPs stimulated apoptosis as exemplified by cell shrinkage, chromatin condensation and nuclear fragmentation, which are markers for apoptosis (Figure 6A–G). While in the control group cells, there is no indication of cell shrinkage, chromatin condensation and nuclear fragmentation (Figure 6A).

As we have seen cancer cell death through MTT assay, we can know that the cause of cancer cell death is due to apoptosis. The DAPI staining showed that 48 h post treatment of MSNPs induced nuclear condensation and cell membrane disruption which are markers for the apoptotic cell death. The control cells, however, remained intact and evenly shaped (Figure 6A). The DAPI staining noticeably showed apoptotic morphological changes in MSNPs-treated cells in terms of both nuclear condensation and cell structure loss.

## 4. Conclusions

The MSNPs were prepared by using hydrothermal method where strontium (Sr), barium (Ba), dysprosium (Dy), samarium (Sm) and iron oxide (Fe_8−2*x*_O_19_) were combined, and dysprosium (Dy) and samarium (Sm) were substituted with strontium (Sr) and barium (Ba), and Sr_0.5_Ba_0.5_Dy*_x_*Sm*_x_*Fe_8−2*x*_O_19_ (0.00 ≤ *x* ≤ 1.0) MSNPs were prepared. The microspheres were characterized by X-ray powder diffraction (XRD), high-resolution transmission electron microscopy (HR-TEM), transmission electron microscopy (TEM), scanning electron microscopy (SEM) and energy-dispersive X-ray spectroscopy (EDX) techniques. The diffraction pattern of NHFs reflected the signature peaks of hexagonal structure. The XRD revealed a pure hexagonal structure without any undesired phase which indicated the homogeneity of the product. The crystal size of the products was valued by Scherrer’s equation in the range of 30 to 36 nm. The SEM of MSNPs showed semi-spherical shape with high degree of aggregation. TEM and HR-TEM images of MSNPs verified the spherical shape morphology and structure that approved the formation of M-type hexaferrite. Anti-cancer activity was examined on HCT-116 (human colorectal carcinoma) and HeLa (cervical cancer cells) using MTT assay, and a post-48 h treatment of MSNPs caused a dose-dependent inhibition of HCT-116 and HeLa cell proliferation and growth. Conversely, no significant cytotoxic effect was observed on HEK-293 cells. The treatments of MSNPs also induced cancer cells DNA disintegration as revealed by DAPI staining. Finally, these findings suggest that synthesized MSNPs possess potential inhibitory actions on cancerous cells without harming normal cells. 

## Data Availability

Data will be available upon request.
